# Elements of the B Cell Signalosome Are Differentially Affected by Mercury Intoxication

**DOI:** 10.1155/2014/239358

**Published:** 2014-05-04

**Authors:** Randall F. Gill, Michael J. McCabe, Allen J. Rosenspire

**Affiliations:** ^1^Department of Immunology and Microbiology, Wayne State University, Detroit, MI 48201, USA; ^2^Department of Environmental Medicine, University of Rochester, Rochester, NY 14642, USA

## Abstract

It has been suggested that environmental exposures to mercury contribute to autoimmune disease. Disruption of BCR signaling is associated with failure of central tolerance and autoimmunity, and we have previously shown that low levels of Hg^2+^ interfere with BCR signaling. In this report we have employed multiparametric phosphoflow cytometry, as well as a novel generalization of the Overton algorithm from one- to two-dimensional unimodal distributions to simultaneously monitor the effect of low level Hg^2+^ intoxication on activation of ERK and several upstream elements of the BCR signaling pathway in WEHI-231 B cells. We have found that, after exposure to low levels of Hg^2+^, only about a third of the cells are sensitive to the metal. For those cells which are sensitive, we confirm our earlier work that activation of ERK is attenuated but now report that Hg^2+^ has little upstream effect on the Btk tyrosine kinase. On the other hand, we find that signaling upstream through the Syk tyrosine kinase is actually augmented, as is upstream activation of the B cell signalosome scaffolding protein BLNK.

## 1. Introduction

Mercury is a potent immunotoxicant. Early experiments demonstrated that exposure to high or moderate levels of mercury induced immune cell death through either necrosis or apoptosis. Fortunately, exposure to high levels of mercury is no longer common, but large segments of the population continue to be exposed to low levels. In contrast to higher level exposures, low level exposure to mercury is not linked to immune cell death, but rather to immune disorders characterized by immunoproliferation and autoimmunity [1, 2].

Today in the US most exposure to mercury is the result of consumption of seafood contaminated with organic mercury. It has also been reported that high fructose corn syrup, a major constituent of most processed foods, often contains surprisingly high levels of inorganic mercury (Hg^2+^), and so low-level Hg^2+^ exposure is now a source of concern [3]. However even with respect to dietary ingestion of organic mercury, the majority is fairly quickly metabolized to Hg^2+^, so that over time mercury burdens in organs such as brain or spleen normally encompass both inorganic and organic species, even under conditions where exposure has been limited to organic mercury [4, 5]. In fact studies in normal individuals, as well as persons known to have been accidentally exposed to high levels of dietary organic mercury, have shown that eventually about 70% of the total mercury burden in spleen and brain is in the form of inorganic mercury [6, 7]. Most importantly, findings show that in mice exposed only to organic mercury, Hg^2+^ produced as a result of the metabolism of organic mercury, but not residual organic Hg, is the mercury species directly responsible for mediating an autoimmune inflammatory response. In other words, with respect to autoimmune disorders and inflammatory processes Hg^2+^ is the active metabolite [8, 9].

In principle, autoimmune disorders might result from a genetic defect in, or environmental interference with the control of central tolerance, the mechanism whereby the immune system neutralizes self-reactive immature lymphocyte clones. In immature B cells the B cell receptor (BCR) complex signaling pathway is of critical importance for this process [10, 11]. In fact it has been shown that there is a direct connection between suppression of BCR signals and induced failure of central tolerance in the B cell compartment leading to increased mature autoimmune B cell clones [12]. The association of exposures to low levels (levels which are not immunosuppressive* in vivo* or trigger immune cell death* in vitro*) of Hg^2+^ with autoimmunity suggests that Hg^2+^ could potentially interfere with signal transduction pathways in immature B cells in a manner so as to compromise central tolerance.

WHEI-231 is a well-characterized mouse B cell line that exhibits many of the characteristics of immature B cells. In particular, in contrast to mature B cells for which stimulation of the BCR is associated with mitogenesis and increased proliferation, WEHI-231 in a process similar to immature B cells undergoes growth arrest and apoptosis in response to BCR stimulation [13, 14]. We have previously shown that exposure of WEHI-231 to low levels of Hg^2+^ interferes with the BCR signaling pathway in triggering growth arrest and apoptosis [15]. Furthermore in immature B cells, growth arrest and apoptosis consequent to BCR signaling are dependent on activation of ERK [16]. We have shown in WEHI-231 that, although ERK does not seem to be a direct target, low level exposure to Hg^2+^ attenuates activation of ERK1/2 during BCR signaling [17], thus suggesting that Hg^2+^ acts on an upstream element of the BCR signal transduction pathway to initially suppress ERK activation and subsequently growth arrest and apoptosis after BCR engagement of an agonist ligand [17].

Phosphoflow cytometry is a relatively new technology which has been shown to be far superior to traditional western blotting technology in investigating phosphorylation dependent signaling pathways in immune cells, and in particular B cells [18–21]. In the experiments outlined below we utilize multiparameter phosphoflow cytometry to simultaneously quantify the activity of several different elements of the BCR signal transduction pathway on a single cell basis. By capturing multiple interrelated phosphorylation patterns in Hg^2+^ intoxicated (exposed) and BCR stimulated WEHI-231 cells we have gained insight into the mechanism whereby Hg^2+^ at low exposure levels interacts with upstream elements of the BCR signal transduction pathway in B cells.

## 2. Materials and Methods

### 2.1. Reagents

HgCl_2_ (≥99.5% pure) was obtained from Aldrich Chemicals (St. Louis, MO).

### 2.2. Cells

WEHI-231 cells were obtained from American Type Culture Collection (Manassas, VA). They are maintained in RPMI 1640 containing L-glutamine (HyClone, Logan, UT) and supplemented with 10% fetal bovine serum (FBS, HyClone), 50 *μ*M 2-mercaptoethanol, and 1X antibiotic antimycotic solution (Mediatech, Herndon, VA) in a humidified 5% CO_2_ atmosphere. Cells were passaged twice a week at a concentration of 1 × 10^5^ cells/mL. Cell viability was monitored visually by Trypan blue exclusion.

### 2.3. Antibodies

Polyclonal goat affinity-purified antibody to mouse immunoglobulin mu chain was purchased from MP-Biomedicals-Cappel, Solon, OH. Fluorescently labeled antibodies used for cytometric analysis of phosphorylated ERK1/2, Syk, BLNK, and Btk were purchased from BD Biosciences (San Jose, CA).

### 2.4. Phosphospecific Flow Cytometry

WEHI-231 cells were counted, washed in serum-free RPMI medium, and resuspended at 3 × 10^6^ cells/mL in the presence or absence of varying concentrations of HgCl_2_ in polypropylene tubes. Subsequently, 1 × 10^6^ cells were added to tubes containing goat anti-mouse immunoglobulin specific to the mu chain. Cells were incubated for the specified period of time at 37°C. At the end of stimulation (0.5, 1, 5, or 10 minutes), the cells were fixed by adding an equal volume of 4% paraformaldehyde, pH 7.4, and incubating for 10 minutes at 37°C. The cells were diluted in Dulbecco's phosphate-buffered saline, pH 7.4, pelleted by centrifugation, resuspended in 90% methanol, and then incubated on ice for at least 30 minutes to permeabilize the plasma membrane. Following permeabilization the cells were pelleted to remove the methanol and then washed twice in sample buffer (Dulbecco's phosphate-buffered saline, 1% FBS, and 0.1% sodium azide). The cells were resuspended in an antibody cocktail containing fluorescently labeled antibodies specific for the phosphorylated residues of ERK1/2, Syk, BLNK, or Btk according to the manufacturer's instructions. The cells were incubated for 1 to 2 hours on ice and then washed 3 times with sample buffer. The cells were kept in the dark at 4°C until being analyzed on a Beckman-Coulter Cyan ADP flow cytometer.

### 2.5. Data Analysis

For all experiments, 10,000 events were acquired per sample and the data were initially analyzed using Sumit (Beckman Coulter; Miami, FL) and/or FlowJo (Tree Star; Ashland, OR) software. Additional data analysis and graphing was done using Excel (Microsoft; Redmond, WA) and Sigma Plot (Systat Software; San Jose, CA) software. For any fluorescently labeled cell population the Mean Fluorescence Index (MFI) is defined as median fluorescence intensity of the population.

## 3. Results and Discussion

### 3.1. Phosphospecific Flow Cytometric Analysis of ERK Activation during BCR Signaling in WEHI-231 B Cells

**Figure 1:**
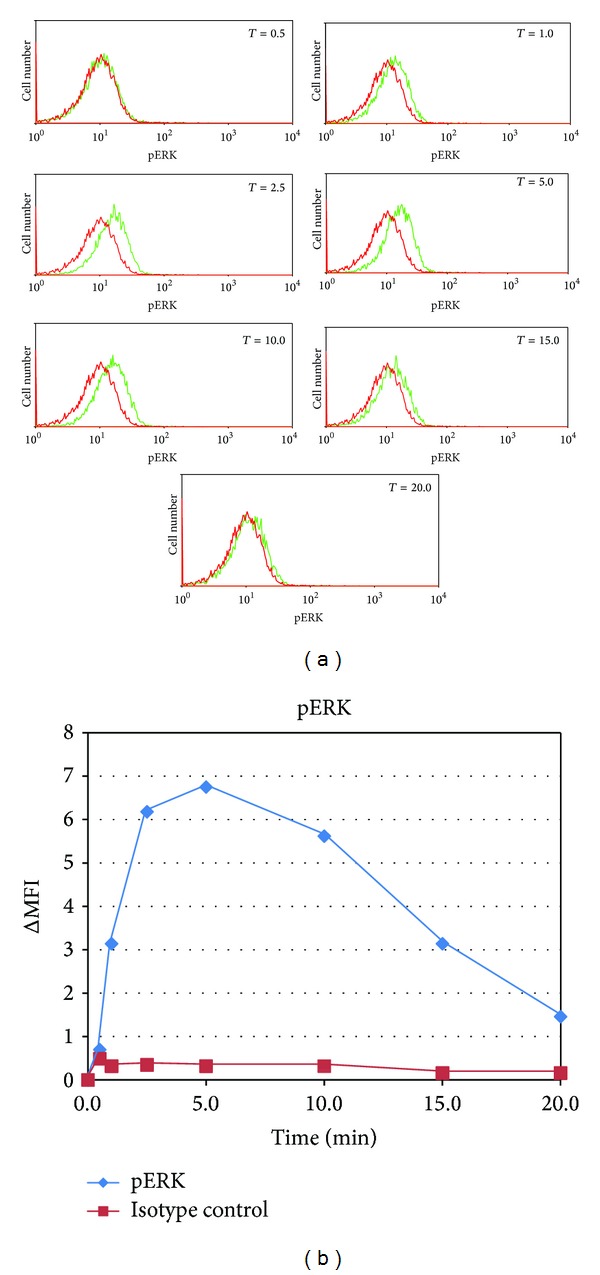
Time course of pERK in WEHI-231 B cells after stimulation of the BCR with anti-mu as determined with phosphoflow cytometry. (a) Histograms at various times for pERK (green, light hue) and isotype control (red, dark hue). (b) Change in MFI (as calculated from the histograms in (a) as a function of time after BCR stimulation.

In our previous investigation of the effect of mercury on BCR signal transduction we employed western blotting technology to show that ERK activation was impaired [17]. However we felt that the application of the newer phosphospecific flow cytometric technology might offer better delineation of the effects of Hg^2+^ on BCR signaling. Accordingly, we first employed phosphospecific flow cytometry to investigate the kinetics of BCR activation of ERK 1/2 in control WEHI-231 cells. ERK1/2 are serine/threonine kinases which in B cells are central to the BCR signal transduction pathway and upon BCR signaling become activated by dual phosphorylation of a canonical threonine/tyrosine motif [22]. In Figure 1, as described in the materials and methods section, WEHI-231 cells were or were not treated with anti-mu (25 *μ*g/mL) to stimulate the BCR. At timed intervals the cells were probed with a fluorescently labeled antibody specific to dually phosphorylated ERK 1/2 (anti-pERK), or else an isotype control antibody. The level of ERK 1/2 activation was then determined by phosphospecific flow cytometry. In Figure 1(a) each of the plots represents a different time after BCR stimulation. In each plot the red line represents the control histogram for which no anti-mu was added, while the green line represents the histogram at that time point arising from BCR activated cells. Initially the two curves overlap and then begin to separate as ERK 1/2 becomes activated in stimulated cells. Maximum separation appears at the 5 minute mark, after which they move together as ERK 1/2 activation is attenuated.

The kinetics of ERK activation resulting from BCR stimulation are perhaps more easily appreciated in Figure 1(b). In Figure 1(b), for each of the distributions where the cells have been probed with anti-pERK (stimulated or unstimulated) shown in Figure 1(a), the Mean Fluorescence Index (MFI), defined as the median fluorescence intensity for that distribution, has been calculated. Then for each time point, the difference between the MFI for the untreated cells and the MFI for the BCR stimulated cells at that time has been plotted. The same procedure was then applied to cells which were probed with the isotype control antibody, and the difference in MFI between anti-mu treated cells and untreated cells plotted.

Generally speaking, because of the large number of cells examined, population differences in MFI determined by flow cytometry tend to be highly significant. For instance the standard error of the median for a normally distributed variable is given by 1.25 $\sigma /\sqrt{n}$, where *σ* is the standard deviation of the distribution, and *n* the number of independent measurements. For the distributions shown in Figure 1(a) (as well as in the other figures in this paper), the *σ*'s are of the order of the MFIs, but since each distribution represents about 10,000 cells, the standard errors are of the order of 1% of the MFIs.

Another way of appreciating the significance of the MFI measurements in Figure 1(b) is to note that, in Figure 1, fluorescence intensity measurements are proportional to the log of the actual fluorescent signal. As a result relatively small changes in the MFI are often significant. By comparing the histograms shown in Figure 1(a) with the MFI differences plotted in Figure 1(b), it is evident that, for all time points between 2.5 and 15 minutes, the histograms of treated cells are significantly different from control cells. In other words, for this experiment, differences of MFI between control and BCR stimulated cells of at least 2 can be considered as meaningful.

**Figure 2:**
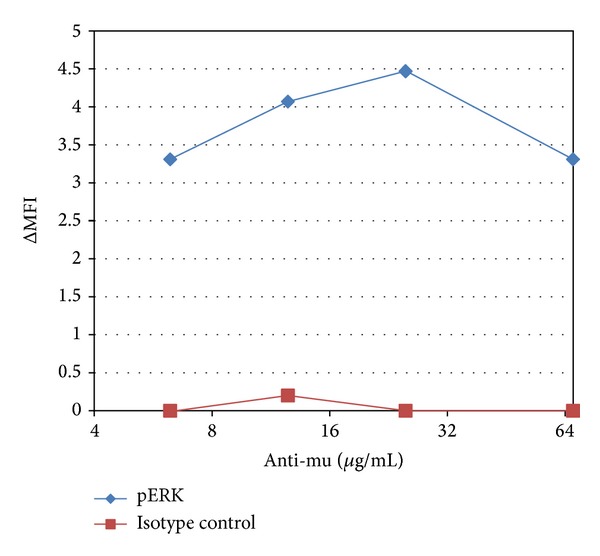
Change in pERK at 5 minutes after stimulation as a function of concentration of anti-mu used to stimulate the BCR.

Figure 1 demonstrates that in WEHI-231 the maximum pERK response to anti-mu stimulation of the BCR occurs in about 5 minutes, after which it trends towards baseline. However in Figure 1 we employed anti-mu at 25 *μ*g/mL to stimulate the BCR. This initial 25 *μ*g/mL figure was based upon our previous experience with this system [17]. However the anti-mu reagent we utilize is a polyclonal antibody, and the potential exists for batch to batch variations in the agonist response. To assure ourselves that we were utilizing an appropriate concentration of stimulating antibody, in Figure 2 we investigated the maximum pERK response at 5 minutes as a function of the stimulating antibody concentration. Accordingly, cells were treated with various concentrations of anti-mu for 5 minutes, and then pERK levels determined by flow cytometry utilizing the anti-pERK reagent used in Figure 1, or an isotype control antibody. pERK and isotype control staining profiles for each concentration of anti-mu were then compared to staining profiles of cells which had not been treated with anti-mu and the difference in MFI was determined and plotted.

### 3.2. Compared to Anti-Mu, Low Concentrations of Hg^2+^ Alone Only Marginally Perturb the Activity of ERK and Syk in the BCR Signaling Pathway

The tyrosine kinase Syk is an important element of the BCR signaling pathway upstream of ERK. During BCR signaling, activation of ERK is known to be dependent upon activation Syk, and, like ERK, Syk activity is increased by tyrosine phosphorylation [22, 23]. In later experiments we will be looking at the effect of Hg^2+^ on ERK and upstream elements of the BCR pathway during signaling, so for control purposes we measured the level of activated ERK and Syk (pERK and pSyk) which was induced by Hg^2+^, alone, in the absence of specific BCR signaling, to those induced by anti-mu through binding to the BCR. Accordingly WEHI-231 cells were treated with (0 *μ*g/mL, 2.5 *μ*g/mL, and 5 *μ*g/mL) Hg^2+^, and, at various time points after addition of Hg^2+^, cells were stained with different fluorescently labeled anti-pERK and anti-pSyk. We have previously shown that treating WEHI-231 cells with concentrations of 5 *μ*g/mL HgCl_2_ for 10 minutes results in cellular burdens of Hg^2+^ of the order of 1 ng/10^7^ cells, and this cellular burden maximally impacts BCR signaling [17]. We have determined by propidium iodide uptake that these* in vitro* Hg^2+^ burdens are not toxic to WEHI-231 cells (NS), while similar* in vivo* Hg^2+^ burdens are not toxic to mouse spenocytes [24]. pERK and pSyk were then simultaneously measured by phosphospecific flow cytometry. At the 10 minute mark anti-mu was added to some of the cells which had not been treated with Hg^2+^. Five minutes later at the 15 minute mark these cells were likewise assayed for pERK and pSyk by phosphospecific flow cytometry. Untreated cells were also labeled with isotype controls to pERK and pSyk antibodies and assayed by flow cytometry.

**Figure 3:**
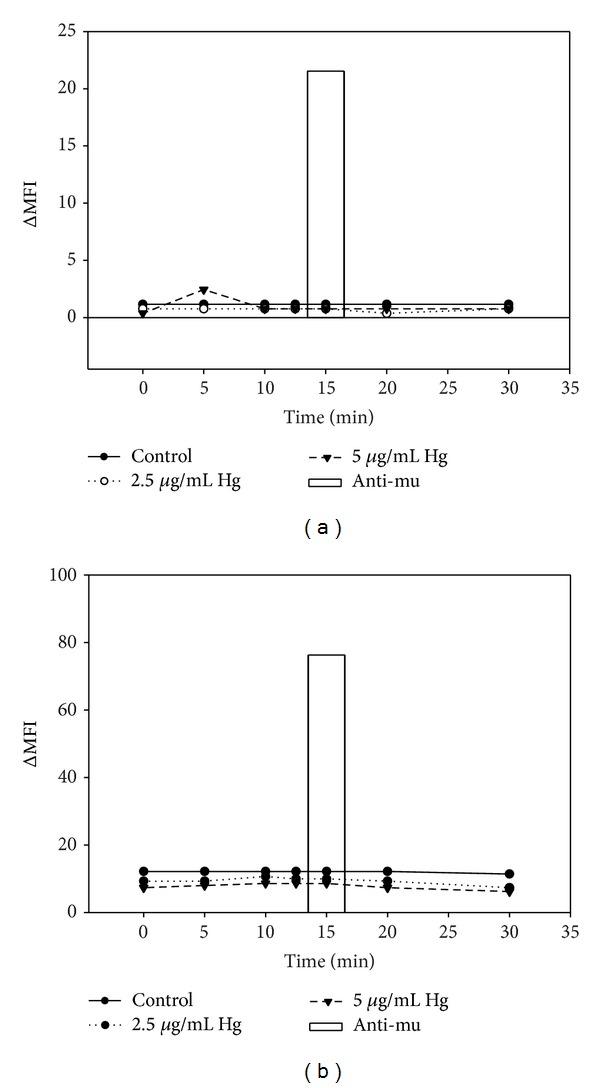
Effect of Hg^+2^ and anti-mu (12.5 *μ*g/mL) as a function of time after addition of (a) pERK and (b) pSyk.

The results for pERK and pSyk are shown in Figures 3(a) and 3(b), respectively, and expressed as the change in MFI between cells which were labeled with the phosphospecific antibodies to those cells which were labeled with the appropriate isotype specific control. The results for the cells which were stimulated with anti-mu are displayed as a single bar plot at the 15 minute mark, to distinguish them from the results for cells which were treated with Hg^2+^, which are displayed as line plots. Compared to anti-mu induced BCR signaling, Hg^2+^ alone has little effect on ERK or Syk.

### 3.3. Low Levels of Hg^2+^ Attenuate Phosphorylation of ERK, Accentuate Phosphorylation of Syk and BLNK, but Has Little Effect on Phosphorylation of BTK during BCR Mediated Signal Transduction in WEHI-231 Cells

We next investigated the effect of Hg^2+^ *on BCR* signaling upstream of ERK. BCR signaling is a complex process that is still not fully understood. However it is generally accepted that shortly after antigen engagement of the BCR complex that multimolecular cluster formation begins and assembly of the B cell receptor signalosome ensues [23, 25]. For the most part the structural integrity of the signalosome is anchored by the scaffolding protein BLNK, whose ability to bind other signalosome constituents is mediated by tyrosine phosphorylation of specific BLNK residues. These residues are substrates of the Btk, Lyn, and Syk kinases, and in an autocatalytic fashion the activity levels of these kinases, particularly Syk, are in turn regulated to some extent by whether or not they are bound to BLNK [23].

**Figure 4:**
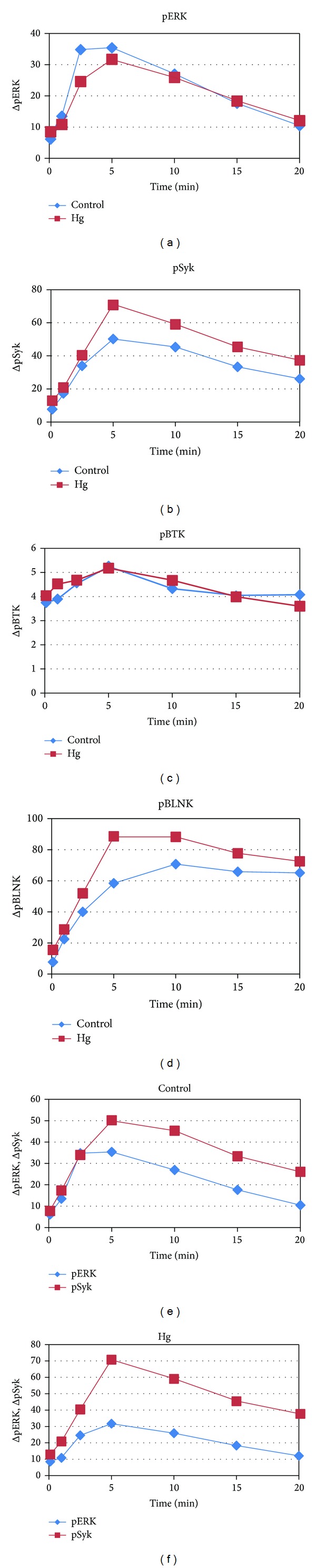
Effect of Hg^+2^ on stimulation of elements of the BCR signal transduction pathway after stimulation of the BCR with anti-mu (12.5 *μ*g/mL). In Figure 4 the* y*-axis represents differences in MFI between (BCR stimulated cells treated or not with Hg^2+^) and control cells which have neither been treated with Hg nor stimulated via the BCR. The* x*-axis represents time after stimulation of the BCR. (a) pERK versus time, (b) pSyk versus time, (c) pBtk versus time, (d) pBLNK versus time, (e) pERK versus pSyk versus time in the absence of Hg^+2^, and (f) pERK versus pSyk in the presence of Hg^+2^.

One of the advantages of phosphospecific flow cytometry is that it provides the ability to simultaneously monitor multiple elements of a phosphorylation dependent signaling pathway in individual cells. Accordingly in Figures 4(a)–4(d) WEHI-231 cells were incubated with or without Hg^2+^ for 5 minutes and then stimulated or not with anti-mu. At timed intervals cells were fixed and levels of activated ERK (pT202/pY204), Syk (pY436), Btk (pY551), and BLNK (pY84) simultaneously determined by multicolor phosphospecific flow cytometry. In each plot cells which have been preincubated with Hg^2+^ are compared to cells which have not. The results are expressed as the difference between the MFI of cells which have or have not been treated with anti-mu as a function of time after addition of anti-mu.

Figure 4(a) demonstrates that Hg^2+^ attenuates the normal ERK signal realized during BCR signal transduction, while Figure 4(b) shows that Hg^2+^ actually enhances the strength of the Syk signal. On the other hand BTK appears unaffected, as Hg^2+^ does not seem to have any appreciable effect on either the magnitude or kinetics of the BTK response. Finally phosphorylation of BLNK, like that of Syk, is enhanced by Hg^2+^.

Although Figures 4(a) and 4(b) show that Hg^2+^ depresses and augments the activation of ERK and Syk, respectively, it is difficult to discern if there are differences in kinetics. To evaluate whether Hg^2+^ altered the kinetics of ERK or Syk activation during BCR signaling we replotted the data of Figures 4(a) and 4(b) in Figures 4(e) and 4(f). In Figure 4(e) we have plotted the change (between cells treated or not with anti-mu) in the MFI of ERK and Syk in (control) cells which have not been pretreated with Hg^2+^. This figure demonstrates that under control conditions the kinetics of ERK activation is in phase with that of Syk activation. In Figure 4(f) we have plotted the same variables; only this time we have used the results from cells which were exposed to Hg^2+^. Here it is clear that although Hg^2+^ may decrease the magnitude (phosphorylation) of the ERK response while increasing the magnitude (phosphorylation) of the Syk response, exposure has little effect on the phase relationship between activation of the two kinases during BCR signal transduction.

### 3.4. Analysis of the Effect of Hg^2+^ on the Activation of ERK and Syk during BCR Signaling on the Single Cell Level

Although we had earlier pointed out that one of the advantages of phosphospecific flow cytometry is that it potentially permits analysis of signaling events on the single level, we did not take advantage of this in the analysis accomplished in Figure 4. In Figure 4 we reduced complex raw data sets obtained from the flow cytometery so as to characterize different cell populations solely with four numbers, the four MFIs associated with binding phosphospecific antibodies to ERK, Syk, BTK and BLNK. The effect of Hg^2+^ on BCR signal transduction was then essentially determined by comparing these numbers among differentially treated cell populations. However the initial data reduction to MFIs means that Figure 4 is in essence a population level analysis, and that similar results could in principle be obtained utilizing western blotting technology, albeit with much greater difficulty and likely less precision.

**Figure 5:**
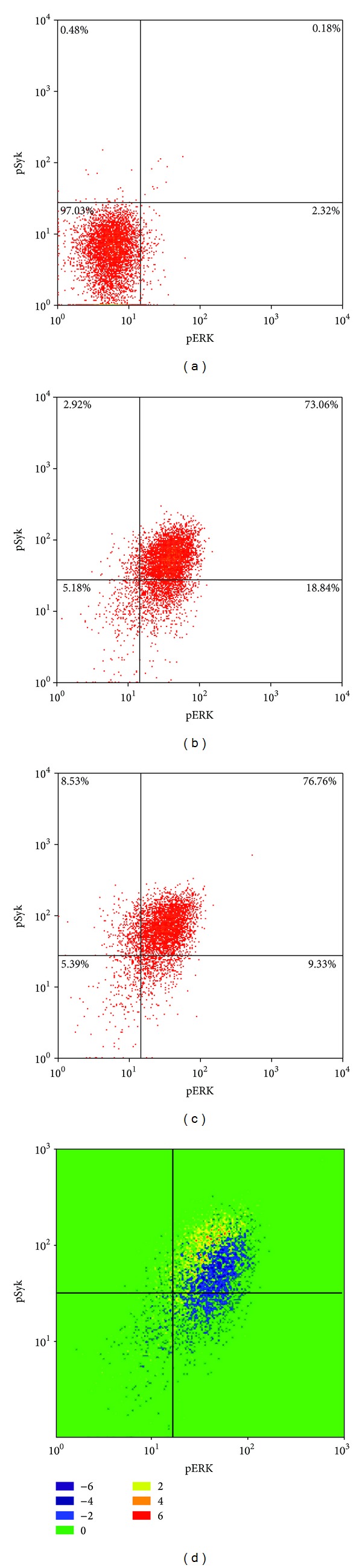
Two-dimensional histogram representation of pSyk versus pERK after stimulation of BCR with anti-mu (12.5 *μ*g/mL). (a) Control cells at 0 minutes after stimulation of the BCR; (b) 5 minutes after stimulation of the BCR; (c) five minutes after BCR stimulation in Hg^+2^ intoxicated cells; (d) “Overton” subtraction of (c) from (b).

In Figure 5 we analyze the effect of Hg^2+^ on ERK and Syk activity during BCR signaling but forego initial reduction to MFIs in order to preserve single cell information. Accordingly Figure 5(a) is a 2-parameter histogram with respect to pSyk (*y*-axis) and pERK (*x*-axis) for the control population of WEHI-231 cells which have not been treated with Hg^2+^ or anti-mu. Here the number of cells expressing any particular pSyk, pERK value pair is proportional to the density of the plot in that region. Since Figure 4 indicated that the maximum response (pERK or pSyk) to anti-mu occurs at 5 minutes after stimulation, in Figure 5(b) we show the 2-parameter (pSyk, pERK) histogram for WEHI-231 cells which have been treated with anti-mu for 5 minutes. Finally Figure 5(c) is the histogram for cells which have been treated with anti-mu for 5 minutes but which have also been pretreated with Hg^2+^. Each histogram is divided into quadrants, and the percent of the cell population falling into each quadrant is indicated.

In Figure 5(a) we have adjusted the quadrant settings so as to characterize 97.03% of the control population as pSyk^low^, pERK^low^ and only 0.18% pSyk^hi^, pERK^hi^. Utilizing the identical quadrant settings, upon anti-mu stimulation this changes after 5 minutes to 73.06% pSyk^hi^, pERK^hi^ and 5.18% pSyk^low^, pERK^low^. As we already determined in Figure 4, when cells are intoxicated with Hg^2+^, the profile of pSyk and pERK is altered after anti-mu stimulation of the BCR from what is found for cells which are not intoxicated. This is reflected in Figure 5(c). After BCR stimulation, for Hg^2+^ intoxicated cells the percentages of cells in each of the quadrants do not drastically differ from those of cells which are not intoxicated, but nevertheless the bulk of the distribution seems to move to higher pSyk and lower pERK values.

Frequently in flow cytometry, cell populations characterized by a single fluorescent intensity are visualized as one-dimensional histograms. In order to analyze shifting population frequencies arising under different experimental conditions the Overton algorithm is often employed to subtract these one-dimensional histograms from one another [26]. In our case we would like to analyze the effect of Hg^2+^ exposure on the population shift that occurs simultaneously with respect to cellular levels of pERK and pSyk after BCR stimulation, and which has been visualized as two-dimensional histograms. Although we are working with two-dimensional histograms, just as for one-dimensional histograms, the effect of Hg^2+^ can be analyzed by subtracting the histogram for nonintoxicated cells (Figure 5(b)) from that for Hg^2+^ intoxicated cells (Figure 5(c)).

To accomplish this subtraction we have generalized the Overton algorithm from one to two dimensions. The result is shown in Figure 5(d) where discrete areas of the plot have been coded according to the legend on the right. Positive numbers indicate areas where there are excessive cell numbers in the Hg^2+^ exposed population with respect to the nonexposed population after BCR stimulation, while negative numbers represent the opposite. The absolute values of the numbers are proportional to the absolute differences, either positive or negative. In the one-dimensional Overton algorithm, the total number of positive cells is summed, and this number divided by the total population number is by definition the percent positive cells. Using an analogous procedure, we find that, in Figure 5(d), 37.6% of the cells in the mercury intoxicated population are designated “positive”.

We have previously shown that intoxication of WEHI-231 B cells with low and nontoxic levels of Hg^2+^ (1 ng/10^7^ cells) has little direct effect on ERK. However, at these levels Hg^2+^ interferes with BCR signal transduction so that activation of ERK is attenuated [17]. These earlier experiments relied upon western blot analysis. The phosphoflow cytometric findings in Figures 1, 3(a), and 4(a) are in complete support of these previous findings. Since there is a direct connection between suppression of BCR signals in immature B cells and increased mature autoimmune B cell clones [12], to the extent that WEHI-231 B cells can be taken as a model of immature B cells, Hg^2+^ attenuation of ERK activation implies that Hg^2+^ intoxication may permit some self-reactive B cell clones to escape central tolerance and contribute to autoimmunity or autoimmune disease upon maturation.

The fact that in both of these experiments and in our earlier work low levels of Hg^2+^ seem to have little direct effect on ERK implies that the target of Hg^2+^ is upstream of ERK, and that signaling through at least some elements of the BCR signal transduction pathway upstream of ERK should also be attenuated. Syk is one such possible element. It is a central tyrosine phosphokinase operating upstream of ERK in the BCR signal transduction pathway, and as such is necessary for the integrity of the BCR signal and the eventual elimination of self-reactive immature B cell clones [22, 23]. Our earlier analysis of Syk activation in Hg^2+^ intoxicated WEHI-231 B cells utilizing western blot technology was inconclusive as to whether Hg^2+^ intoxication attenuated Syk activation [17]. However utilization of phosphoflow cytometry (Figure 4(b)) now shows quite clearly that Hg^2+^ enhances the activation of Syk.

Aside from Syk, we have also looked at phosphorylation of BLNK and Btk, two other elements upstream of ERK in the BCR signal transduction pathway. BLNK is the scaffolding protein that is important for the structural integrity of the B cell signalosome, which forms consequent to antigen engagement of the BCR [23, 25]. During BCR signaling, phosphorylation of various BLNK tyrosine residues by the tyrosine kinases Syk, Btk, and Lyn is necessary for the proper formation and maturation of the signalosome, as various signalosome constituents specifically recognize and bind to these phosphorylated motifs (primarily through SH2 domains) [25]. As Btk activation is itself associated with the phosphorylation of tyrosine 551 [27], both BLNK and Btk activities were assayed by phosphoflow cytometry to determine the effect of Hg^2+^ intoxication on their activity during BCR signaling (Figures 4(c) and 4(d)). We find that while Hg^2+^ has little effect on Btk activation during BCR signaling, similar to Syk, phosphorylation of BLNK is significantly enhanced. Considering that BLNK is a substrate of Syk, this was expected.

On the other hand, the finding that Syk activation is enhanced, while downstream activation of ERK is attenuated by Hg^2+^ was a bit surprising, especially considering our earlier work with T cells. BCR signaling has much in common with T Cell Receptor (TCR) signaling. In particular, in T cells TCR cross-linking leads to the transient activation of ERK, much like BCR cross-linking leads to the activation of ERK in B cells. We have found that in T cells intoxicated with low levels of Hg^2+^ ERK activation is attenuated during TCR signaling [28]. In T cells, TCR dependent ERK activation is dependent upon upstream activation of the phosphotyrosine kinase ZAP-70, a homologue of Syk. However in T cells we have found that attenuation of ERK by Hg^2+^ is associated with attenuation of ZAP-70, as well as LAT, the scaffolding protein homolog of BLNK [29].

While Figures 4(c) and 4(d) show that during BCR signaling, Hg^2+^ dependent attenuation of ERK activation is correlated with enhancement of Syk activation, these experiments only speak of what is happening on a population level. The question remains whether this correlation strictly holds on the cellular level. Utilizing the identical dataset used to generate Figure 4, Figures 5(a)–5(d) specifically take advantage of the ability of phosphoflow cytometry to simultaneously probe individual cells for Syk and ERK activity to answer this question. Figure 5(a) is the two-dimensional histogram plotting pSyk versus pERK activity levels in control cells, while Figure 5(b) plots pSyk versus pERK activity in cells which have been maximally stimulated with anti-IgM. Comparison of Figures 5(a) and 5(b) demonstrates that most cells (about 90%) respond to BCR stimulation by increasing Syk and ERK activity.

Figure 5(c) is the pSyk versus pERK histogram for cells which have been stimulated with anti-IgM as in Figure 5(b), but which have also been intoxicated with Hg^2+^. Again, most cells still seem to be responding to anti-IgM as in Figure 5(b), but the distribution appears to be subtlety shifted to lower pERK and higher pSyk levels. This of course is consistent with Figure 4 demonstrating that on a population level Hg^2+^ intoxication leads on average to measurably lower pERK and higher pSyk levels. To assess the effect of Hg^2+^ intoxication on BCR signaling on the cellular level, we have utilized a generalized Overton algorithm to subtract the histogram in Figure 5(b) from that of Figure 5(c), resulting in Figure 5(d).

When one-dimensional histograms are subtracted it is common practice to display only the one-dimensional histogram of the resulting positive cells. However for the two-dimensional subtracted histogram in Figure 5(d) we display positive as well as negative areas. We interpret positive areas of the histogram (which are represented in brighter hues) as cellular positions which become more populated in Hg^2+^ intoxicated populations after BCR signal induction than in control populations. Likewise the negative areas of the histogram (represented by darker hues) represent cellular positions which become less populated as a result of Hg^2+^ intoxication. They essentially represent areas of the histogram from which cells (in the control) will move from as a result of Hg^2+^ exposure.

In Figure 5(d), the positive and negative areas are well segregated, with the positive areas having higher pSyk, but lower pERK, values. This finding supports the notion that on a cellular as well as population level, in Hg^2+^ intoxicated cells suppressed ERK activity is associated with enhanced Syk activity. Significantly, in Figure 5(d) we find that 37.6% of the cells are “positive.” In this context we interpret positive to mean that only 37.6% of Hg^2+^ intoxicated cells respond to BCR cross-linking with depressed ERK and enhanced Syk activation when compared to control cells. The remaining 62.4% of Hg^2+^ intoxicated WEHI-231 cells respond to BCR cross-linking by enhancing pERK and pSyk, just as they would as if they had not been exposed to Hg^2+^.

The overall finding that Hg^2+^ augments activation of Syk while it simultaneously attenuates downstream activation of ERK is at first glance a bit puzzling. However a recent report suggests that in B cells there are two functionally and topologically distinct ERK activation pathways [16]. Although both depend on Syk as an upstream element, they diverge shortly afterward. The first one is the familiar pathway previously described for B cells [22, 23], but it is postulated that this pathway is primarily operable in mature cells. Activation of this pathway is presumed to initiate cell proliferation. A second ERK activation pathway is described and postulated to be primary active in immature B cells. This pathway is connected to apoptosis and clonal deletion. The major difference between the two pathways is that this second pathway depends on a BCR mediated Icrac calcium current. If Hg^2+^ was to depress the calcium signal by interfering with an upstream regulator of Icrac channels, then it would be possible that ERK activation through this pathway could still be attenuated, even though upstream signaling through Syk was augmented. In any event it seems clear that Hg^2+^ initially interacts with a B cell target or targets upstream of Syk, possibly including Icrac channels.

## 4. Conclusions

After exposure of WEHI-231 B cells to low levels of Hg^2+^, signal transduction initiated by the BCR is altered. Analysis on the single cell level shows that while activation of ERK is suppressed, upstream activation of Syk is enhanced and phosphorylation of BLNK is increased. On the other hand, activation of Btk is unaffected. As WEHI-231 is a model for immature B cells, suppression of ERK activation in these cells suggests that low level exposures to Hg^2+^ may also interfere with central tolerance in primary immature B cells. We suggest that interference with central tolerance may be one of the mechanisms connecting low level mercury intoxication to autoimmune disease.
